# NeSSM: A Next-Generation Sequencing Simulator for Metagenomics

**DOI:** 10.1371/journal.pone.0075448

**Published:** 2013-10-04

**Authors:** Ben Jia, Liming Xuan, Kaiye Cai, Zhiqiang Hu, Liangxiao Ma, Chaochun Wei

**Affiliations:** 1 School of Biomedical Engineering, Shanghai Jiao Tong University, Shanghai, China; 2 Department of Bioinformatics and Biostatistics, School of Life Sciences and Biotechnology, Shanghai Jiao Tong University, Shanghai, China; 3 School of Bioengineering, East China University of Science and Technology, Shanghai, China; 4 Shanghai Center for Bioinformation Technology, Shanghai, China; Belgian Nuclear Research Centre SCK/CEN, Belgium

## Abstract

**Background:**

Metagenomics can reveal the vast majority of microbes that have been missed by traditional cultivation-based methods. Due to its extremely wide range of application areas, fast metagenome sequencing simulation systems with high fidelity are in great demand to facilitate the development and comparison of metagenomics analysis tools.

**Results:**

We present here a customizable metagenome simulation system: NeSSM (Next-generation Sequencing Simulator for Metagenomics). Combining complete genomes currently available, a community composition table, and sequencing parameters, it can simulate metagenome sequencing better than existing systems. Sequencing error models based on the explicit distribution of errors at each base and sequencing coverage bias are incorporated in the simulation. In order to improve the fidelity of simulation, tools are provided by NeSSM to estimate the sequencing error models, sequencing coverage bias and the community composition directly from existing metagenome sequencing data. Currently, NeSSM supports single-end and pair-end sequencing for both 454 and Illumina platforms. In addition, a GPU (graphics processing units) version of NeSSM is also developed to accelerate the simulation. By comparing the simulated sequencing data from NeSSM with experimental metagenome sequencing data, we have demonstrated that NeSSM performs better in many aspects than existing popular metagenome simulators, such as MetaSim, GemSIM and Grinder. The GPU version of NeSSM is more than one-order of magnitude faster than MetaSim.

**Conclusions:**

NeSSM is a fast simulation system for high-throughput metagenome sequencing. It can be helpful to develop tools and evaluate strategies for metagenomics analysis and it’s freely available for academic users at http://cbb.sjtu.edu.cn/~ccwei/pub/software/NeSSM.php.

## Introduction

Metagenomics studies all genetic materials recovered directly from environmental samples. It is a new research area full of promising since it can provide insight to a large unexplored field of uncultured microbes, while traditional isolate-and-culture methods can only analyze a tiny fraction of total microbes [1], [2]. For example, metagenomics methods have been applied to analyze numerous microbial communities [3] and new organisms or enzymes have been found by using metagenomics methods [4], [5]. High-throughput sequencing technologies play a key role here, especially for shotgun sequencing-based metagenomics analysis [6], [7]. Over all, these new sequencing platforms, which are called Next-Generation Sequencing (NGS) technologies in general, have revolutionized the sequencing landscape in the past a few years [8], [9]. Due to their high throughput and cost-performance efficiency, NGS platforms have made it possible to apply metagenomics methods to a wide range of research areas.

In order to reduce the resources, cost and time for a metagenomics project, it’s better to evaluate and compare the sequencing strategies in advance [10]. This is particularly important when applying a metagenomics method to a new research area. A metagenome sequencing simulation system can be helpful for this purpose [11]. Besides, it can help develop subsequent analysis tools, such as assembly and classification [12], [13]. In addition, since no gold standard is available for metagenomic data analysis, performance evaluation based on simulated metagenome sequencing is still the most effective way [14].

In NGS sequencing simulation, several important technical specifications of the NGS sequencing platforms need to be considered. First, the sequencing errors should be simulated since it can be critical to the subsequent analysis. For example, a better microbial diversity is extracted when the sequencing errors in 454 sequencing platforms are considered [15]. The sequencing errors vary a lot for different NGS platforms. For example, in Illumina platform, mismatches happen more frequently than indels, while it is opposite in 454 platform [16]. Second, sequencing coverage bias should be considered as well. In both Illumina and 454 platforms, the bases in a genome are not sequenced equally [17], which is defined as sequencing coverage bias. Sequencing coverage bias can also affect subsequent analysis, such as community diversity estimation [18]. Therefore, it is necessary to simulate both the sequencing errors and sequencing coverage bias for metagenome sequencing simulations. At the mean time, the simulation of metagenome data is becoming more time consuming since the throughput of the NGS sequencing platforms is huge. For instance, the latest version of Illumina HiSeq 2000 can produce more than 600 G bps in a single run. Therefore, the simulation speed should be considered as well.

Sequenced genomes are required for metagenome sequencing simulation. During the past a few years, the number of sequenced microbial genomes has increased quickly. Particularly, the human microbiome project (HMP) planed to complete and deposit more than 1,000 bacterial genomes and 375 had been completed and deposited in 2009 [19]. There are 1,080 bacterial genomes deposited up to Dec 2, 2012 (http://www.hmpdacc.org/HMRGD/). In addition, phylogenetic tree driven sequencing projects have created genomes distributed more evenly across the polygenetic tree, which makes the microbial genome database much more representative [20]. Overall, there are 2,358 of bacterial and archaeal species with complete genomes available in NCBI genome database on Apr. 8, 2013 (ftp://ftp.ncbi.nih.gov/genomes/).

Currently, there exist some simulation systems for NGS sequencing. Some systems, like ART [21] and pIRS [22], are designed for single genome sequencing or re-sequencing simulation. In recent years, MetaSim, GemSIM and Grinder [16], [23], [24] are developed to facilitate the development of metagenomics analysis systems. MetaSim is a popular simulator for metagenome sequencing and in a single run it uses fixed probabilities of sequencing errors (insertions, deletions and substitutions) for the same base in different reads. GemSIM can estimate the error model from the sequenced datasets. Grinder can simulate PCR amplification and shotgun sequencing with a specific community structure, but its error models are similar to MetaSim’s. However, neither MetaSim nor Grinder takes sequencing coverage bias into consideration, and their sequencing quality values are fixed at the same positions of all reads.

In this paper, we first introduce the Next-generation Sequencing Simulator for Metagenomics (NeSSM). In its error model, each position contains an explicit distribution of sequencing quality values. Sequencing coverage bias can be considered in NeSSM. In addition, it can obtain metagenomic community compositions from existing sequencing data, which can be helpful to improve the fidelity of metagenome simulation. Subsequently, the performance of NeSSM is evaluated. Finally, NeSSM and several existing simulation systems such as MetaSim, GemSIM and Grinder are compared.

## Materials and Methods

### Database of Complete Microbial Genomes

NeSSM contains a database of complete microbe genomes currently available. Bacterial genome sequences (together with archaeal genome sequences) were downloaded from NCBI genome database (ftp://ftp.ncbi.nih.gov/genomes/). Up to the date Apr. 8, 2013, this dataset contained genomes from 2,358 species with a size of 7.8 GB.

### The System Diagram of NeSSM

**Figure 1:**
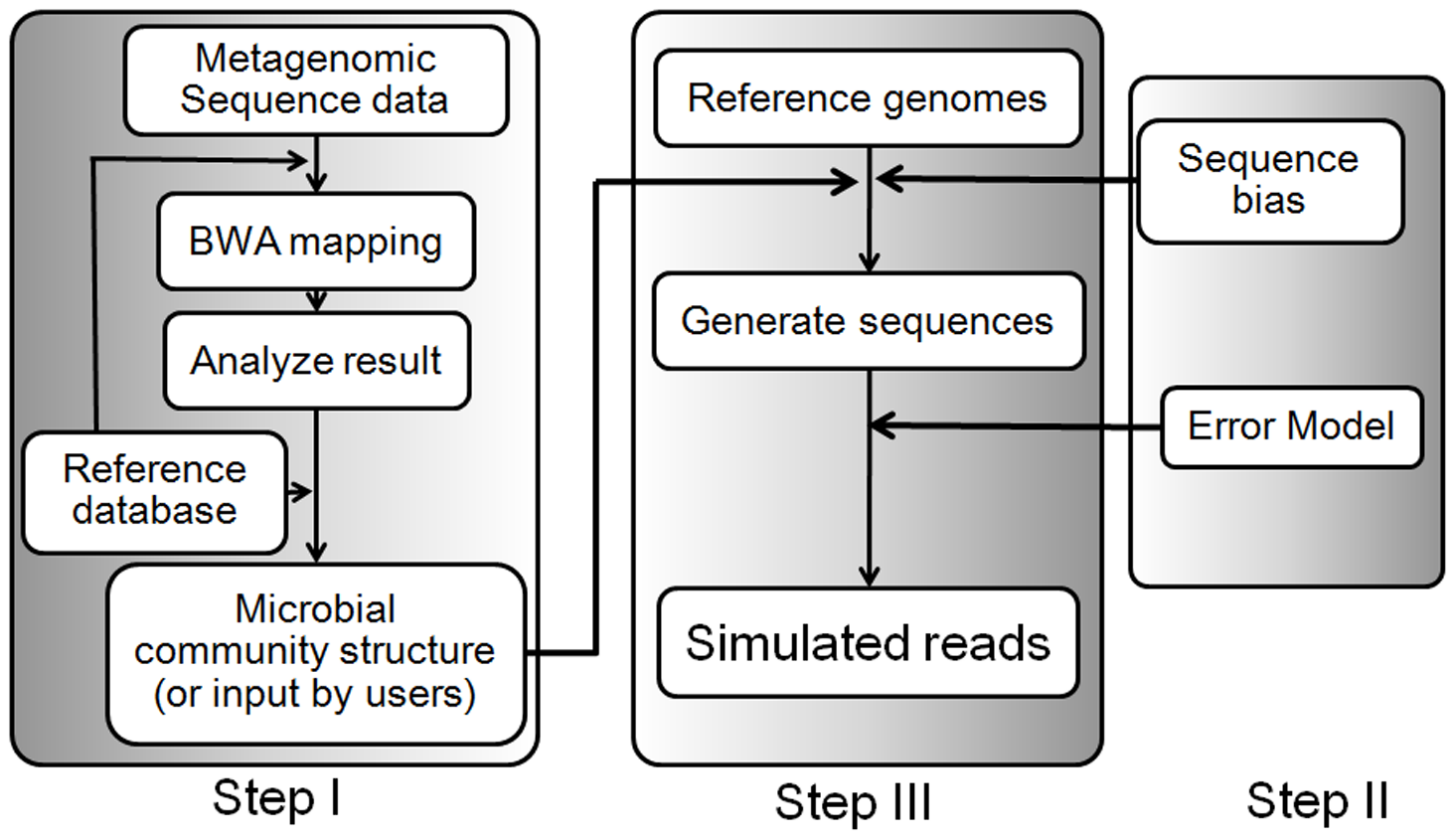
The pipeline of NeSSM system. Step I: extraction of community composition from metagenome sequencing data. This step can be skipped if users have the community composition table already. Step II: estimation of sequence error model and sequencing coverage bias information. Step III: sequencing simulation.

As shown in Figure 1, NeSSM system can be divided into three steps: I, community composition extraction; II, error models and sequencing coverage bias estimation; and III, sequencing simulation. Step III is the main part of the system. Step I and II can be skipped if the corresponding information is available.

#### Step I: community composition extraction

NeSSM takes input files in one of the two types: 1, a table of community composition, which contains a list of microbes and their corresponding proportions; 2, metagenomic sequence data in FASTA or FASTQ format. Step I is required only for the latter input type. The quality control (QC) processes such as removing adaptors, contaminations and low-quality reads are also required in step I in order to get better performance. Details for step I are described below.

First, the metagenome sequencing reads are mapped back to the database of complete microbial genomes with BWA, a popular sequence mapping tool [25]. Different algorithms of BWA are adopted according to the read lengths. For reads shorter than 200 bps, the BWA options are “-I -N” in “aln” step and “-n 100” in “samse/sampe” step; and for reads longer than 200 bps, the option “bwtsw” is used.

Second, the mapping results are analyzed to generate the number of reads for each genome, from which an initial community composition of the metagenome will be calculated. Pseudo-counts (0.001 by default) can be used for genomes. If a read can be mapped to one genome only, the read number for this genome will be added by one. However, if a read can be mapped to multiple genomes, the read counts for those mapped genomes will be increased with weights according to the following equation.
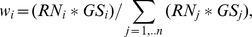
where 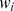 is the weight for genome *i*; 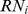 is the number of reads assigned to genome *i*; 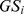 is the size of genome *i*; 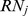 is the number of reads assigned to genome *j*; 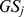 is the size of genome *j*; and *n* is the total number of mapped genomes for this read.

After all reads are assigned, the total number of reads assigned to each genome is obtained. Genomes with read numbers greater than a certain threshold (10 by default) will be added into the candidate list. Then the proportions of all genomes in the candidate list will be calculated according to their read counts.

In the end of step I, the resulted community composition is saved in a table containing the genome names and their abundances.

#### Step II: error models and sequencing coverage bias estimation

Users can set their own error models, which can come from specific publications or be estimated from an existing sequencing dataset. The existing sequencing dataset can be either an initial sequencing dataset or a publicly available sequencing dataset, from the same platform as the user plans to use. Perl scripts for estimating error models from existing sequencing data are provided. The pipeline is described as follows.

First, the sequencing error probability at each base is estimated from the sequencing data according to the quality values. For each base in the FASTQ file, the error probability *p* is calculated from the quality value *Q* through the PHRED score [26], [27]:

PHRED = −10*log(*p*);

*Q* = PHRED+64 (for Illumina platforms);

*Q* = PHRED+33 (for 454 platforms).

**Figure 2:**
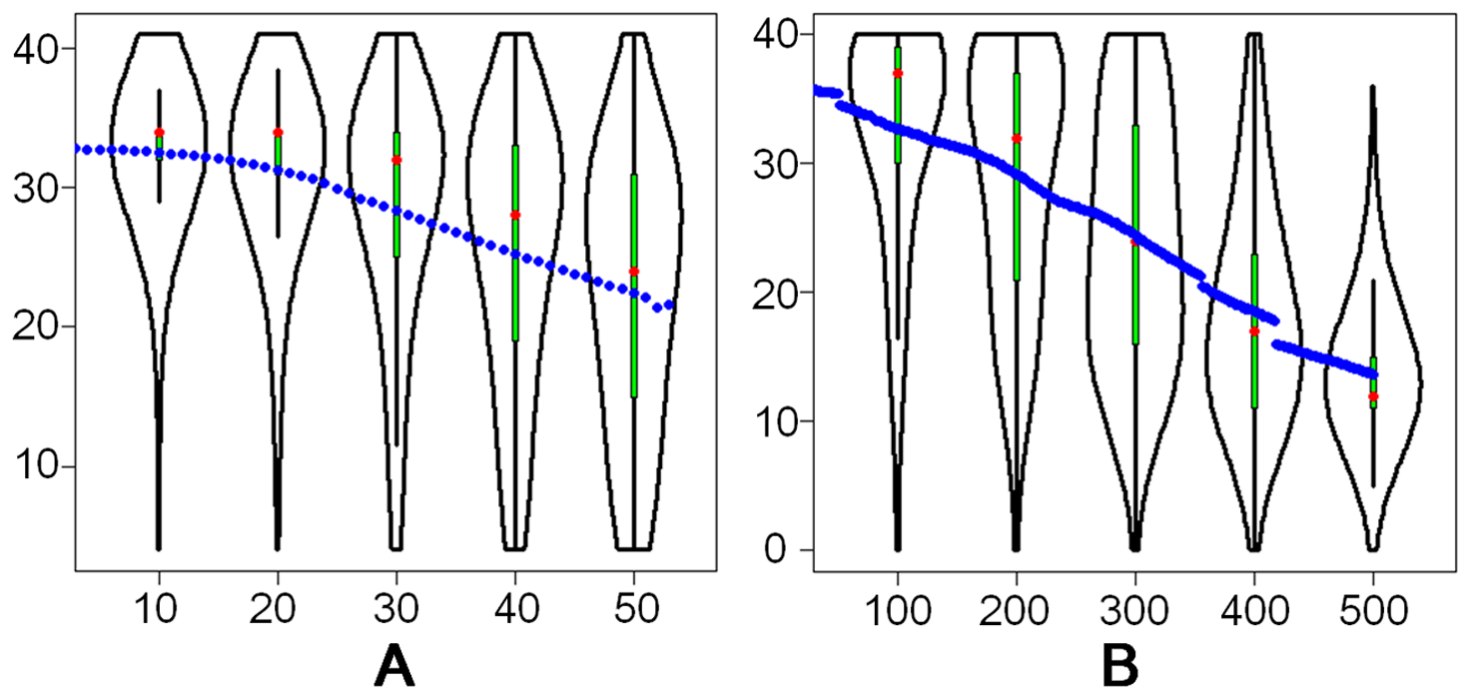
The distribution of quality values at each base. X axis: the coordinates of reads (0-based); Y axis: the PHRED scores. The blue dots represent the average quality values and the red dots represent the median. In each picture, distributions of quality values at five different bases are shown as examples: (A) the distributions of quality values from an Illumina sequencing dataset; and (B) the distributions of quality values from a 454 sequencing dataset. Both datasets contain experimental sequencing data from the sequence read archives of NCBI. See Table S1 for details of the datasets. This figure is plotted by vioplot [42].

It is shown that sequencing quality values at each base follow non-standard distributions (Figure 2 and Figure S1) and the distributions vary for different bases as well. Therefore, explicit distribution, rather than an average value, is utilized to represent the errors at each base in NeSSM simulation.

**Figure 3:**
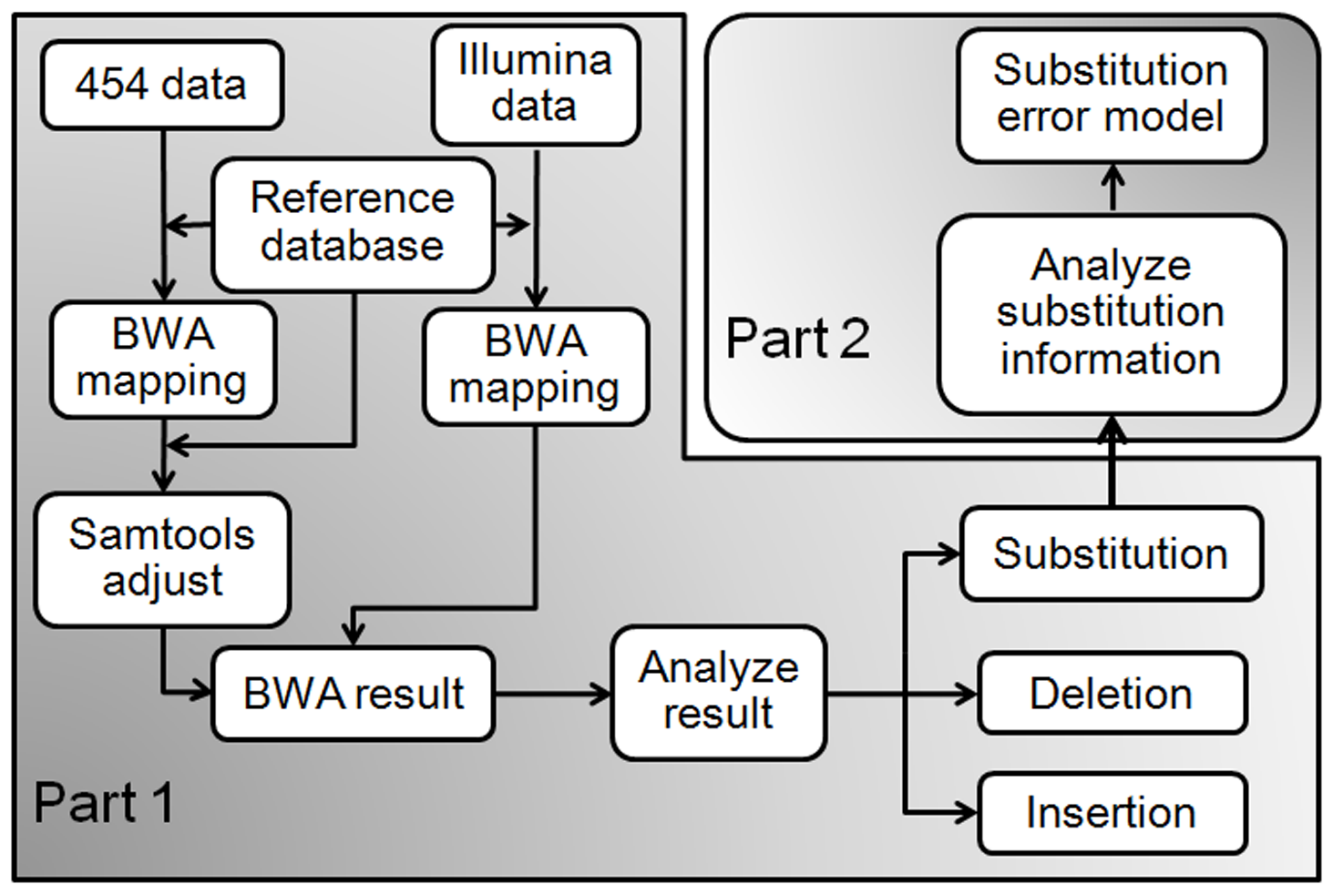
The pipeline of error model estimation. Estimation of the error model can be divided into two parts: 1. Estimation of the proportions of substitutions, insertions and deletions; 2. Estimation of proportions of different type of substitutions.

Subsequently, the proportions of different error types (substitutions, insertions, and deletions) are estimated. This part can be further divided into two parts (Figure 3): first, proportions of substitutions, insertions, and deletions are estimated; second, proportions of different substitutions are calculated.

In order to count the numbers of different error types, mapping results from step I will be adjusted using Samtools [28] if reads (used in the mapping) are greater than 200 bps. Next, information of MD (Mismatching positions/bases), NM (Editing distance) and CIGAR (alignment information) are extracted from the mapping results.

Then, the proportions of insertions, deletions and sum of different kinds of substitutions are calculated from the counts of NM and CIGAR. For Illumina sequencing, the default error proportions are set to 0.01, 0.005 and 0.985 for insertion, deletion and substitution, respectively; for 454 sequencing, the default error proportions are set to 0.2, 0.15 and 0.65 for insertion, deletion and substitution, respectively. All the default parameters are estimated from sequencing data listed in Table S1.

At the end, the proportions of different kinds of substitutions can be calculated from MD and CIGAR values. Table 1 shows the substitution errors for 454 sequencing. The substitution errors for Illumina sequencing are listed in Table S2.

**Table 1:** The proportions of substitution errors used in 454 sequencing simulation.

Substitute Real	A	T	C	G
A		0.06474	0.05735	0.12439
T	0.05378		0.13480	0.06577
C	0.05298	0.11709		0.06752
G	0.12384	0.06784	0.06990	

**Figure 4:**
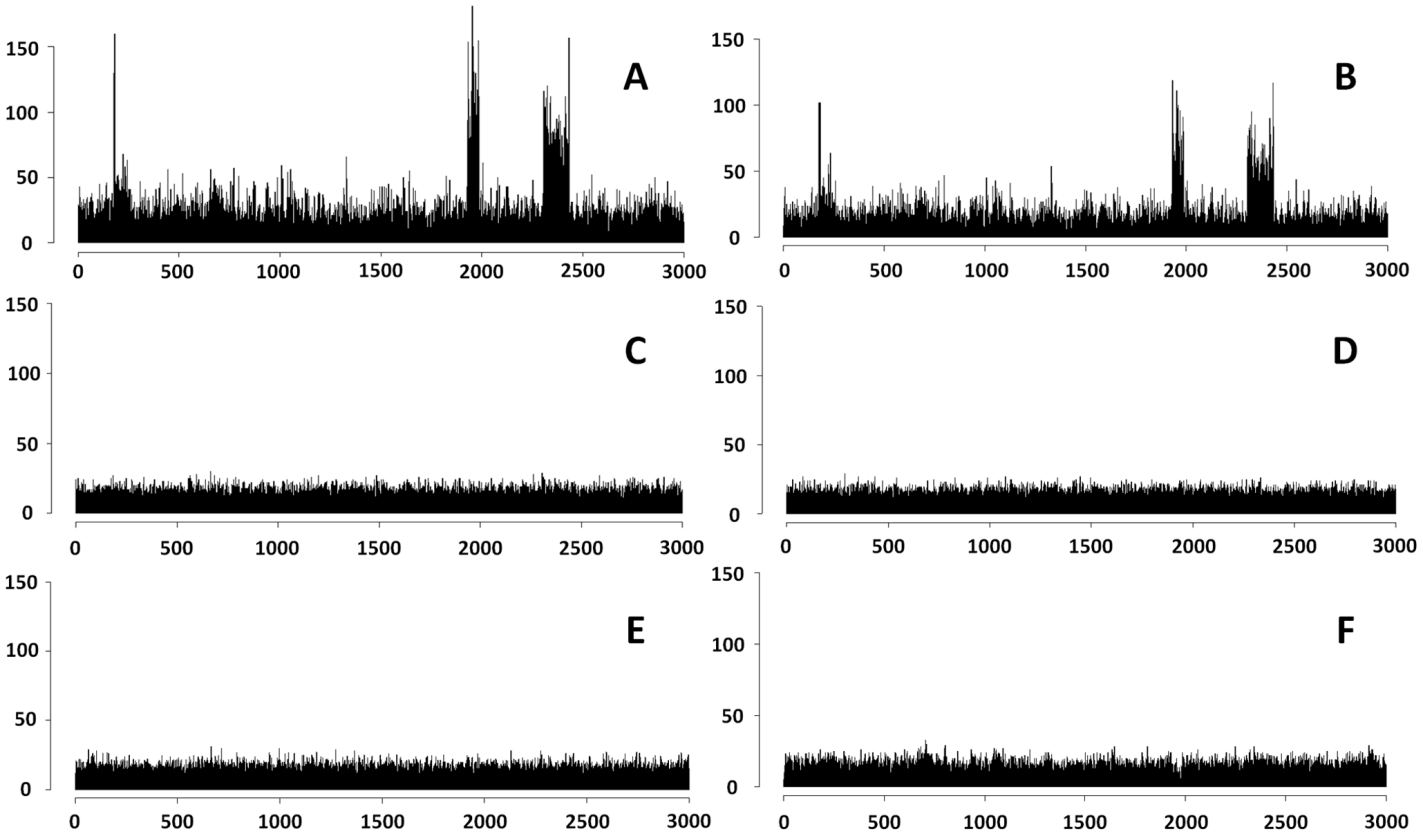
The comparison of sequencing coverage before and after simulation. X axis: the coordinate of the genome of Acinetobacter baumannii ATCC 17978. Each interval contains 100 bases and only the first 3,000 intervals are shown; Y axis: the read numbers mapped in each interval. A: the sequencing coverage in the Dataset F; B: the sequencing coverage in NeSSM’s simulation; C: the sequencing coverage in MetaSim’s simulation; D: the sequencing coverage in GemSIM’s simulation; E: the sequencing coverage in Grinder’s simulation; and F: the sequencing coverage in pIRS’s simulation.

The sequencing coverage bias information can be extracted from an existing metagenome sequencing dataset. First, each metagenome read is mapped back to its genome using BWA with parameters described in the above section. Next, all genomes are divided into intervals of 100 bps. One mapped read adds 1 count to the corresponding interval. In addition, an initial pseudo-count (0.1) is given to each interval. Finally, the sequencing coverage of the genome is calculated based on the read counts for the intervals (more details in Figure 4).

#### Step III: sequencing simulation

In the sequencing simulation step, the number of reads for each genome is first determined. Then reads are generated for each genome. Simulation details are described below.

The expected read number for each genome is determined according to its abundance and genome size. For example, if the composition structure table contains two genomes: A and B, with abundances of 0.4 and 0.6 respectively, and their genome sizes of 15 M bps and 10 M bps respectively, then the expected read numbers for these two genomes are the same. If the total number of simulated reads is 2 million, the expected read number will be 1 million for each genome. In practice, the read number of each genome is simulated according to a multinomial distribution with the probability of every genome set as the ratio of the expected number of reads and the total number of reads.

After the number of reads for each genome is determined, reads are generated from the selected microbial genome. First, according to the sequencing parameters provided by the user, the exact read is simulated as follows. For each read, a coordinate in the corresponding genome is generated randomly with even distribution or according to the sequencing coverage bias if it is provided; then the read length is determined according to the read length distribution; at last a piece of sequence exact to its origin genome is generated. The read length distribution can be of fixed length, normal distribution or explicit length distribution. Second, errors, including substitution and indel errors, are added to the sequence to generate the final read. In order to add errors, first, it should be decided whether an error or errors will be added. If an error or errors will be added, it should be decided what kind(s) of error(s) should be added according to the error models.

### GPU Version of NeSSM

Graphics processing units (GPUs) are originally designed to accelerate graphic display only. In the past few years, GPUs have evolved to GPGPUs (general purpose GPUs) and provide a massive parallel platform for scientific and engineering computing. Next-generation sequencing data analysis is a good application field for GPUs since millions of reads need to be processed in the same way, which can be parallelized easily. GPUs have been used in the bioinformatics area, including a popular sequence mapping program, SOAP3 [29], a metagenomics analysis program, Parallel-META [30], and MetaBinG [31], a fast metagenomic classification program. A GPU version of NeSSM based on CUDA (Compute Unified Device Architecture) is also developed to accelerate the simulation. It works as follows.

First, the number of reads to be simulated for each genome is decided on CPUs. Then the corresponding genome sequence and its read number are loaded to GPUs. The sequencing simulation process is similar as in CPUs except that every 20,000 reads (by default) are generated and output from GPUs in a batch. If the number of reads to be generated is smaller than 20,000, only the required number of reads are output from GPUs.

### Datasets

In order to assess the performance of NeSSM, six datasets (Dataset A to F) with different complexity are obtained. Three of the six datasets are simulated metagenomes: Dataset A, low complexity dataset (LC); Dataset B, median complexity dataset (MC); and Dataset C, high complexity dataset (HC). A single genome sequencing dataset from Illumina platform (Dataset D) is used to evaluate error models and distributions of quality values in NeSSM’s simulation. A 454 sequencing dataset from an artificial metagenome (Dataset E) and an Illumina sequencing dataset from a mock metagenome in the HMP project (Dataset F) are used to compare the performance of NeSSM and existing simulators such as MetaSim, GemSIM, and Grinder. Details for these datasets are shown below.

#### Dataset A: LC dataset

The Dataset A contains two microbial species with one dominant species. It is also called the dataset with low complexity (LC dataset). Details are listed in Table S3.

#### Dataset B: MC dataset

The Dataset B contains nine microbial species divided into five abundance levels, with two dominant genomes. It is also called the dataset with medium complexity (MC dataset). Details are listed in Table S3.

#### Dataset C: HC dataset

The Dataset C contains eleven microbial species with the same relative abundance for all species. It is also called the dataset with high complexity (HC dataset). Details are listed in Table S3.

#### Dataset D: A single genome illumina sequencing dataset

Dataset D contains Illumina sequencing reads for a single genome downloaded from SRA (run number SRR524810) in NCBI. It contains 4,353,518 reads. After QC (see more details bellow), 1,890,277 reads with lengths of 120 bps are obtained.

#### Dataset E: An artificial metagenome by 454 sequencing platform

Dataset E is an *in vitro* simulated metagenome [32] (SRR033549 from SRA in NCBI). In this artificial metagenome, Morgan *et al*. mixed 10 microbial species with equal numbers of cells. Subsequently, the DNA of the metagenome was extracted and sequenced by 454 platform. At last, 505,962 reads with an average read length of 243 bps were obtained. After QC (see more details bellow), 475,694 reads with an average length of 193 bps are remained.

#### Dataset F: An artificial metagenome dataset by illumina sequencing platform

At the start of HMP project, a synthetic mock community of 21 known organisms was used to evaluate different protocols (http://www.hmpdacc.org/HMMC/). This dataset (SRR172902 from SRA in NCBI) contained 6,562,065 reads with lengths of 75 bps (Illumina platforms). Three organisms in the community have no complete genomes available in NCBI, but only contigs (more details in Table S4). After QC (see more details bellow), reads from these organisms are removed according to the BWA result and 2,975,345 reads are remained.

### QC for Sequencing Datasets D, E and F

The program IlluQC.pl in NGSQCToolkit [33] has been adopted to do the quality control. For Dataset D and F, the parameters are “-se 6 4–l 90”. In order to reduce the effect of “N”, only the first 120 bps of each read are retained for Dataset D. For Dataset E**,** the first 4 bps bases are deleted and the “Ns” in the ends are also deleted. After that, the last 50 bps of each read are removed because of primers. Finally, only the reads with length more than 60 bps are retained in Dataset E.

### Measuring the Difference between Two Datasets

BC-distance [34] is adopted to measure the difference between two vectors. Let *X* and *Y* be two vectors with *n* dimensions, the BC-distance between *X* and *Y* can be calculated as below.
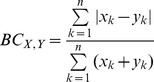
where 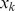 and 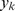 are the k^th^ dimension values of X and Y respectively, 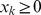, 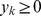 and 
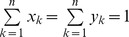
.

In this paper, the BC-distance has been applied to measure the degree of similarity of community composition, error rates and sequencing coverage between a simulated data and an experimental data. For metagenome community composition comparison, X represents the composition table from the simulated data and Y represents the composition table from the other data. For sequencing errors between the simulated sequences and experimental sequences, X represents the error ratios of insertion, deletion and substitution from simulated data and Y represents those from existing data. The BC-distance has also been applied to measure the difference between sequencing coverage of each genome in a simulated metagenome sequencing data and an experimental data. In this case, each genome is divided into an array of windows with a certain size. The average number of reads per window is counted and normalized as the element of X and Y.

### Machine Configurations

All simulations listed in this paper are carried out on a CentOS 5.8 Linux machine with Xeon e7-4807 CPU, 64 GB memory and an NVIDIA Tesla C2050 GPU.

## Results

### The Performance of NeSSM

In order to evaluate the accuracy of NeSSM’s simulation, simulated reads were mapped to the genomes from which they were generated. For Dataset A, B, and C, two types of 454 sequencing reads were simulated with different parameter settings: 150,000 reads with an average read length of 100 bps, and 60,000 reads with an average read length of 250 bps. In total, 6 datasets were simulated. After simulating, Blast [35] was utilized to map those reads back to genomes with default parameters. Next, MEGAN [36] was applied to check how many reads can be mapped back correctly to the genomes where they were generated. The rates of successfully mapped reads were calculated. As a result, most simulated reads could be mapped to their reference genomes, and a small proportion of simulated reads couldn’t be mapped back due to the sequencing errors (Table 2). It demonstrated preliminarily that NeSSM could simulate metagenomic data with necessary sequencing errors and with fidelity as well. Results of simulation with Illumina platform was similar (data not shown).

**Table 2:** The proportions of unique, not unique and not hit reads for 454 simulation datasets from NeSSM.

	read number	% unique	% not unique	% not hit
LC-100	150,000	99.936	0	0.064
LC-250	60,000	100	0	0
MC-100	150,000	78.116	21.597	0.287*
MC-250	60,000	79.158	20.83	0.012
HC-100	150,000	99.299	0.008	0.693*
HC-250	60,000	99.977	0	0.023

Unique: the read is mapped back only to its original genome; Not unique: the read is mapped back to more than one reference genomes; Not hit: the read can’t be mapped back to any reference genome. LC-100 is the simulated data from the low complexity metagenome (Dataset A) with read length 100 bps and other datasets are named similarly for simulated data derived from Dataset B and C.

* most reads of not hit are because of the repeats in the reads so that they can’t be mapped back uniquely by Blast.

**Figure 5:**
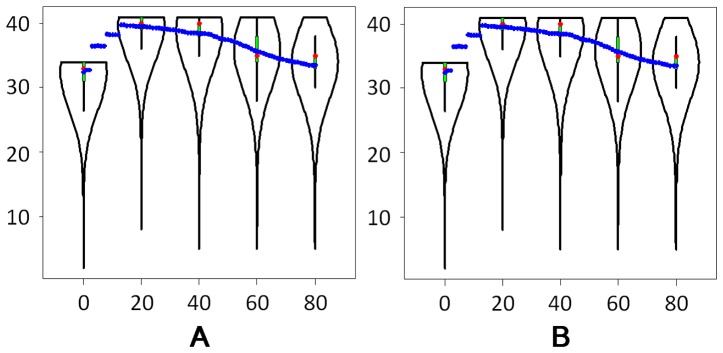
The comparison of distributions of quality values before and after simulation. A: the distributions of PHRED score from the dataset D at five different coordinates; B: the distributions of PHRED score in NeSSM’s simulation. The meaning of blue and red dots is the same as in Figure 2.

Next, NeSSM’s ability to estimate and enforce the error models was evaluated using Dataset D. Error models were derived from Dataset D. These error models were then used by NeSSM to generate simulated reads. First we checked the total number of sequencing errors from the simulated data. The number of sequencing errors per read was 0.1461, which was consistent with the value derived from Dataset D directly (0.1452 errors per read). Next, the proportions of different type of errors (insertions, deletions and substitutions) were further investigated. As a result, the ratios of insertions, deletions and substitutions were 0.00498, 0.00985 and 0.98518 respectively in the simulation, and the ratios were 0.00489, 0.00997 and 0.98514 respectively in dataset D and their BC distance (see Methods part for more details) was 0.00012. In addition, substitution rates were also consistent with those estimated from Dataset D directly (Table 3). Subsequently, the distributions of quality values were also evaluated based on the same simulated data and Dataset D. We showed that the distribution of quality values at each base didn’t follow a normal distribution (Figure 5A), nor was it fixed. In order to conquer this issue, an explicit distribution at each base was utilized in NeSSM and the simulation was consistent well with Dataset D (Figure 5A and 5B). It demonstrated NeSSM could simulate NGS sequencing reasonably.

**Table 3:** The comparison of the proportions of different kinds of substitutions before and after simulation.

	A	T	C	G
A	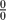	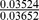	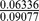	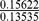
T	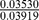	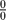	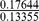	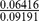
C	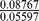	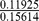	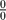	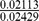
G	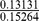	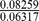	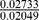	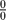

The values above the black lines are estimated from Dataset D and the values below are calculated from NeSSM’s simulation. For example, the row of base “A” shows probabilities of substitutions errors from A to other bases.

**Figure 6:**
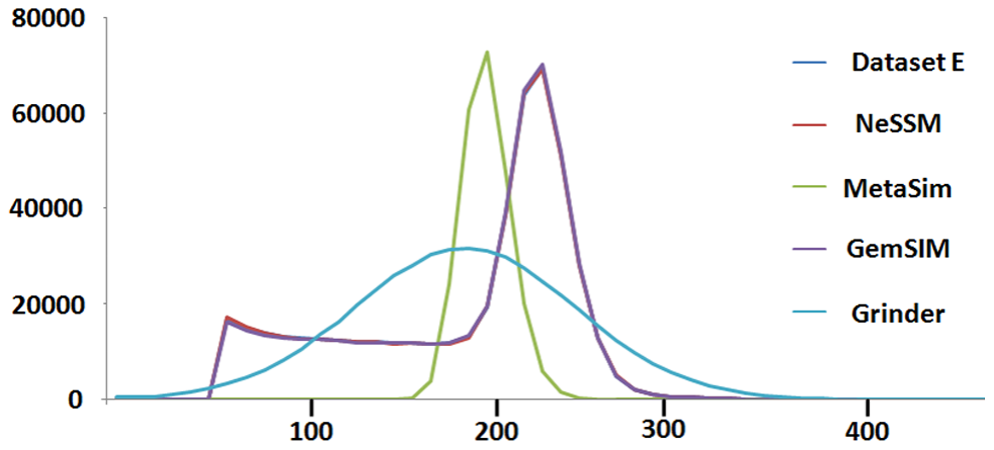
The distributions of read lengths. X axis: the lengths of reads. Each interval is 10 bps. For example, every read with length from 100 bps to 109 bps is counted to the bin of 100 bps; Y axis: the number of reads with lengths in a certain interval. The distributions in NeSSM and GemSIM are close to the actual distribution in Dataset E.

Thirdly, we showed why explicit distribution was required for read length simulation, especially for 454 sequencing platform. Figure 6 showed the distribution of reads length in the Dataset E (for 454 platform). The read length distribution in Dataset E was not a fixed length distribution, not a uniform distribution and not a normal distribution as well. Therefore, explicit length distribution was required for Dataset E and NeSSM was able to simulate reads with an arbitrary length distribution.

Finally, NeSSM’s ability to simulate sequencing coverage bias was evaluated. The composition structure table, error models and information of sequencing coverage bias were estimated from a mock data with 21 known organisms (Dataset F). Then, NeSSM simulated a metagenome. (Like in Dataset D, the ratios of insertions, deletions and substitutions were 0.0359, 0.0171 and 0.9470 respectively in the simulation, and the ratios were 0.0361, 0.0171 and 0.9468 respectively in dataset F. The BC distance of sequencing errors was 0.0002. In addition, substitutions rates were also consistent with those estimated from Dataset F directly (Table S5).) By mapping reads of Dataset F back to their reference genomes, we found that Acinetobacter baumannii (ATCC 17978) held the largest number of reads (Table S4). The sequencing coverage of the Acinetobacter baumannii in the simulation was compared with that in Dataset F. As shown in Figure 4A, the bias of sequencing coverage was significant in Dataset F. The sequencing coverage of the simulated reads was shown in Figure 4B. The BC distance and the correlation coefficient between the sequencing coverage of Acinetobacter baumannii in simulated data and Dataset F were 0.093 and 0.950 (calculated from the first 3,000 intervals), respectively. (The BC distances and the correlation coefficients between the sequencing coverage of other genomes in the simulated data and Dataset F were listed in Table S6.) Therefore, we demonstrated that NeSSM could simulate sequencing coverage bias. If there is no existing sequencing dataset available to estimate the sequencing coverage bias of related genomes, users can remove the NeSSM option “-b” to switch off the “sequencing coverage bias” correction feature. Some existing systems, such as pIRS [22], could simulate the sequencing coverage bias. However, pIRS only simulated pair-end reads and it was not designed for metagenome simulation. In order to test its performance in sequencing coverage bias, Acinetobacter baumannii (ATCC 17978) was simulated using pIRS with 606,771 reads (according to Table S4 and the total reads in Dataset F). The error model was evaluated from Dataset F with scripts supplied by pIRS and only the forward reads in pair-end reads were considered. The result was in Figure 4F. NeSSM had a better performance than pIRS.

### Comparison of NeSSM and Existing Simulation Systems

NeSSM, MetaSim, GemSIM and Grinder were compared in terms of accuracy and speed. The accuracy evaluation was based on the fidelity of the simulation as well as the assembly results of simulations. The comparisons of the assembly of the simulations, the length distribution of reads and sequencing coverage bias are described below.

First, MetaSim and Grinder utilized fixed probabilities to describe the errors at each base in different reads, while NeSSM and GemSIM utilized explicit distributions, which was more consistent with real sequencing data. Second, MetaSim, GemSIM and Grinder simulated all bases from a genome equally (Figure 4), while NeSSM could incorporate the sequencing coverage bias. Besides, NeSSM and GemSIM could simulate reads based on parameters estimated from an existing sequencing dataset. Moreover, NeSSM can extract the community composition from the sequencing dataset, which was quite useful. More comparison was in Table 4.

**Table 4:** The comparison with existing simulation systems.

Systems	NeSSM	MetaSim	GemSIM	Grinder	pIRS
**Sequencing** **platform**	454Illumina	Sanger454Illumina	454Illumina	Sanger454Illumina	Illumina
**Language**	C, PerlCUDA C	Java	Python	Perl	C++Perl
**Single or** **pair-end**	Both	Both	Both	Both	Pair-end
**Error** **Types**	IndelSubstitutionCoverage bias	IndelSubstitutionHomopolymer	IndelSubstitutionSNP	IndelSubstitutionHomopolymer	IndelSubstitutionCoverage bias
**Length** **Distribution**	FixedNormalExplicit	FixedNormal	FixedExplicit	FixedNormalUniform	Fixed
**Output file**	FASTQ	FASTA	FASTQ	FASTAFASTQ	FASTQ
**Quality value** **fixed** *	No	Yes	No	Yes	No
**Application** **Field**	Metagenome	Metagenome	Metagenome	MetagenomeAmplicon	Single genome
**Estimating error models** #	Yes	No	Yes	No	Yes
**Operating system**	Linux	LinuxWindowsMac	Linux	Linux	Linux

* In a sequencing run, whether the quality value is fixed or not for the same positions in different reads;

# Whether the system supplies tools or not to estimate error models from an existing sequencing data.

The speed of NeSSM, MetaSim, GemSIM and Grinder was compared by simulating 90,000,000 reads on both Illumina platform (with read lengths of 36 bps) and 454 platform (with an average read length of 250 bps). NeSSM’s CPU version was more than 2.5 times faster than MetaSim and NeSSM’s GPU version was more than 18 times faster than MetaSim (Table 5). The speed of GemSIM and Grinder was very slow even compared to that of MetaSim. Therefore, NeSSM could be very efficient to simulate large size datasets, especially when GPU card was available.

**Table 5:** Comparison of the speed of NeSSM (CPU and GPU versions) and existing tools on HC metagenome simulation.

Software	Platform	Read number	Read length	Time(s)
**NeSSM_CPU**	Illumina	90 million	36	763
**NeSSM_GPU**	Illumina	90 million	36	200
**MetaSim**	Illumina	90 million	36	3,821
**GemSIM** *	Illumina	90 million	36	90,600*
**Grinder** *	Illumina	90 million	36	2,143,078*
**NeSSM_CPU**	454	90 million	250	5,560
**NeSSM_GPU**	454	90 million	250	773
**MetaSim**	454	90 million	250	13,968
**GemSIM** *	454	90 million	250	631,359*
**Grinder** *	454	90 million	250	2,236,412*

* predicted by a linear extension of the times for a series of small datasets.

The assembly results of NeSSM, MetaSim, GemSIM and Grinder simulation were also compared for both 454 platforms and Illumina platforms.

For 454 platforms, Dataset E was used as a standard. First, with the community composition provided by *Morgan et al.*, 4 metagenomes were simulated by NeSSM, MetaSim, GemSIM and Grinder. Second, a re-estimated community composition table was generated from Dataset E using NeSSM. In order to investigate the impact of the re-estimation of community composition and compare different systems, other 4 metagenomes were simulated by NeSSM, MetaSim, GemSIM and Grinder with the re-estimated community composition table. In all simulations, MetaSim and Grinder used their own error models. NeSSM and GemSIM used the error models estimated from Dataset E. In order to be comparable, 475,694 reads (with average read lengths of 193 bps) were simulated for each dataset. Subsequently, these datasets were assembled by SOAPdenovo [37] with default parameters and statistics based on contigs with lengths greater than 300 bps were compared. The statistics of the contigs included 1, numbers of contigs; 2, average lengths of contigs; 3, N50s and 4, maximum lengths of contigs. The distributions of read length were also compared for those datasets generated by simulation systems (Figure 6).

The re-estimated community composition was similar to the original one supplied by Morgan *et al*. (Table S7). The BC distance between the two community compositions was 0.02593. It demonstrated that community composition estimation system of NeSSM was reasonably effective. Subsequently, 8 simulated metagenomes were assembled by SOAPdenovo. Results of the assembly showed that metagenomic data simulated by NeSSM were much closer to Dataset E than those simulated by MetaSim, GemSIM and Grinder (Table 6 and S8). In addition, the performance of MetaSim, GemSIM, Grinder and NeSSM using the re-estimated community composition was comparable to those using the original community composition. It provided further evidence that community composition estimation system of NeSSM was effective.

**Table 6:** Comparison of NeSSM and existing tools (MetaSim, GemSIM and Grinders).

	Number(contig length> = 300 bps)	Max contig length(bps)	Average contig length(bps)	N50(bps)
**Dataset E**	24,999	6,358	709	810
**NeSSM** #	23,541	5,712	756	881
NeSSM*	23,660	5,905	764	894
**MetaSim** #	10,578	1,124	391	378
MetaSim*	10,792	1,204	390	379
**GemSIM** #	23,665	2,680	498	504
GemSIM*	23,932	3,111	501	507
**Grinder** #	21,648	2,247	459	457
Grinder*	21,876	2,425	463	462

**Simulations are based on Dataset E, an artificial 454 metagenome.** The number, average length and N50 of contigs are all calculated based on contigs longer than 300 bps.

# The community composition was supplied by Morgan et al; and * the community composition was re-estimated by NeSSM.

For Illumina platforms, NeSSM, MetaSim, GemSIM and Grinder were compared based on Dataset F (see Methods for details). First, a community composition was estimated with NeSSM. MetaSim and Grinder used their own error models. NeSSM and GemSIM used the estimated error models from Dataset F. In order to be comparable, 2,975,345 reads with length 75 bps were simulated by the four systems. SOAPdenovo with the default parameters was used to assemble the simulated datasets and Dataset F to evaluate the fidelity of simulation. The results showed that NeSSM simulated metagenomes better than MetaSim, GemSIM and Grinder (Table 7 and Table S9).

**Table 7:** Comparison of NeSSM and existing tools (MetaSim, GemSIM and Grinder) on Dataset F.

	Number(contig length> = 200 bps)	Max contig length(bps)	Average contig length(bps)	N50(bps)
**Dataset F**	37,011	26,131	554	707
**NeSSM**	36,686	53,469	551	685
**MetaSim**	33,720	12,184	627	921
**GemSIM**	32,119	46,796	728	1,350
**Grinder**	32,321	58,173	733	1,391

The number, average and N50 are all based on those contigs of more than 200 bps.

### Using NeSSM to Evaluate Different Metagenome Assembly Methods

NeSSM could be used to evaluate metagenome sequencing data analysis tools. Two assembly systems, SOAPdenovo and MetaVelvet [38] (based on Velvet [39]), were evaluated. MetaVelvet was developed to assemble metagenomic data. However, SOAPdenovo was originally developed to assemble single genome.

Several simulated datasets were created by NeSSM to evaluate two assembly systems. 3 million, 24 million and 30 million reads from Illumina sequencing platform with read length of 36 bps were simulated for Dataset A, B and C, respectively.

The results of assembly were shown in Table 8. Although MetaVelvet was created originally to assemble metagenomes and SOAPdenovo for single genomes, in most cases, the metagenome assembly results from SOAPdenovo were similar with MetaVelvet. Both SOAPdenovo and MetaVelvet got some contigs longer than 10,000 bps.

**Table 8:** Evaluation of assembly tools SOAPdenovo and MetaVelvet using simulation datasets.

		LC	MC	HC
**Number** (contig> = 100 bps)	SOAPdenovo	1,324	51,387	52,710
	MetaVelvet	1,042	55,497	55,369
**Max contig length**(bps)	SOAPdenovo	25,860	51,815	17,004
	MetaVelvet	25,859	46,391	18,570
**Average contig length**(bps)	SOAPdenovo	1,875	427	824
	MetaVelvet	2,343	400	787
**N50**(bps)	SOAPdenovo	6,361	1,366	1,279
	MetaVelvet	6,170	1,051	1,223

The number, average and N50 are all based on those contigs of more than 100 bps.

## Discussion

Evaluating the performance of methods for metagenome sequence analysis is an interesting problem with a large number of audiences. Simulation is still the most effective way to do it. However, simulating metagenome sequencing is not a straightforward task. Here we present a simulation system for next-generation sequencing of metagenomes. It has four highlights:

1. The community composition of a metagenome can be estimated from existing metagenomic data, which will make the simulated metagenome sequencing data more similar to the experimental metagenome sequencing data.
2. Sequencing errors with an explicit distribution at each base are incorporated in the simulation, and they can be estimated from some initial sequencing results.
3. Sequencing coverage bias may be also incorporated in the simulation.
4. NeSSM is a fast tool for metagenome sequencing simulation. GPU version of NeSSM is more than one-order of magnitude faster than MetaSim.

One of the systems similar to NeSSM is MetaSim. It provides a graphic interface for users. However, it does not provide a tool to simulate reads from existing metagenomic data and its error models are not flexible enough to represent the distribution of errors at each base, no sequencing quality value is simulated and the sequencing coverage bias is not fully covered in MetaSim. Other systems similar to NeSSM are GemSIM and Grinder. Their features were listed and compared in Table 4.

NeSSM is useful for development of tools in metagenomics and has been successfully used in the development of an ultra-fast metagenomic sequence classification system, MetaBinG [31].

There are numerous metagenomics projects on going now. However, many questions exist. How deep should a metagenome be sequenced? What sequencing technologies (platforms) should be used? Which data analysis tool(s) should be used? And for selected tools, what values should be set for the parameters? It is difficult to answer any of these questions without simulations. Currently, people’s answers to these questions depend on their experiences rather than on calculation. It is time-consuming and cost-intensive to try and go experimentally. Our system adds one better option to test strategies of complicated metagenomics analysis based on simulations. Combining with other metagenomic analysis tools, NeSSM can be applied to determine some critical parameters for these projects. For example, if metagenome sequencing methods is applied to get the full genome sequence of an uncultured microbe, the minimum coverage of this genome or the number of reads for this genome is a critical parameter [40], and it may be decided by simulations followed by subsequent analysis.

However, NeSSM has limits, especially, for some samples where the number of complete genomes currently available is not enough to simulate a reasonable metagenome. For example, there are very few complete genomes that can be mapped with the shotgun sequencing data from some environments such as the rumen. Consequently, it’s hard to simulate a reasonable metagenome for those environments. In addition, only the organisms with complete genomes can be put in the reference database for the current version of NeSSM. In the near future, NeSSM should be extended to include organisms with contigs only in the reference database. Besides, the information of sequencing coverage bias can only be estimated from existing sequencing data using script provided by NeSSM. There are evidences that the sequence biases are affected by G+C content. The simulator pIRS uses the G+C content to simulate sequencing coverage [41], but the correlation is not very strong. Therefore, NeSSM still uses an explicit distribution for sequencing coverage bias.

## Conclusions

In this paper, we present NeSSM, a fast Next-generation Sequencing Simulator for Metagenome sequencing with customizable community composition and estimated sequencing error models. Overall, NeSSM can be helpful to develop tools and to determine some critical parameters for projects based on metagenome sequencing.

### Availability and Requirements

The NeSSM system is available freely for academic users at http://cbb.sjtu.edu.cn/~ccwei/pub/software/NeSSM.php. The simulated datasets and parameters used in this paper are also provided in this website. It was implemented with C programming language, CUDA (GPU version 4.0 or up) and Perl (5.8 or higher) in a Linux operating system.

## Supporting Information

Figure S1 The distributions of quality values at each base plotted by vioplot.
(TIF)

Table S1 A complete list of public datasets used in this paper.
(XLSX)

Table S2 The proportions of substitution errors used in Illumina sequencing simulation.
(DOCX)

Table S3 A complete list of genomes in LC, MC and HC datasets.
(XLSX)

Table S4 Details of the mock Dataset F: species and their abundance estimated by NeSSM.
(XLSX)

Table S5 The comparison of the proportions of different kinds of substitutions before and after simulation in Dataset F. The layout of this table is similar to that of Table 3.
(DOCX)

Table S6 The correlation coefficient and BC distance between the sequencing coverage bias of each genome in Dataset F and that in the simulated data.
(XLSX)

Table S7 The community composition together with the abundance supplied by Morgan *et al.* and NeSSM.
(XLSX)

Table S8 Comparison of assembly of NeSSM’s and existing tools’ (MetaSim, GemSIM and Grinder’s) simulation based on Dataset E.
(XLSX)

Table S9 Comparison of assembly of NeSSM’s and existing tools’ (MetaSim, GemSIM and Grinder’s) simulation based on Dataset F.
(XLSX)
