# Supplementary figures and images for: NeSSM: A Next-Generation Sequencing Simulator for Metagenomics
Source: PLoS One. 2013 Oct 4;8(10):e75448. doi: 10.1371/journal.pone.0075448 (PMC3790878; doi:10.1371/journal.pone.0075448)

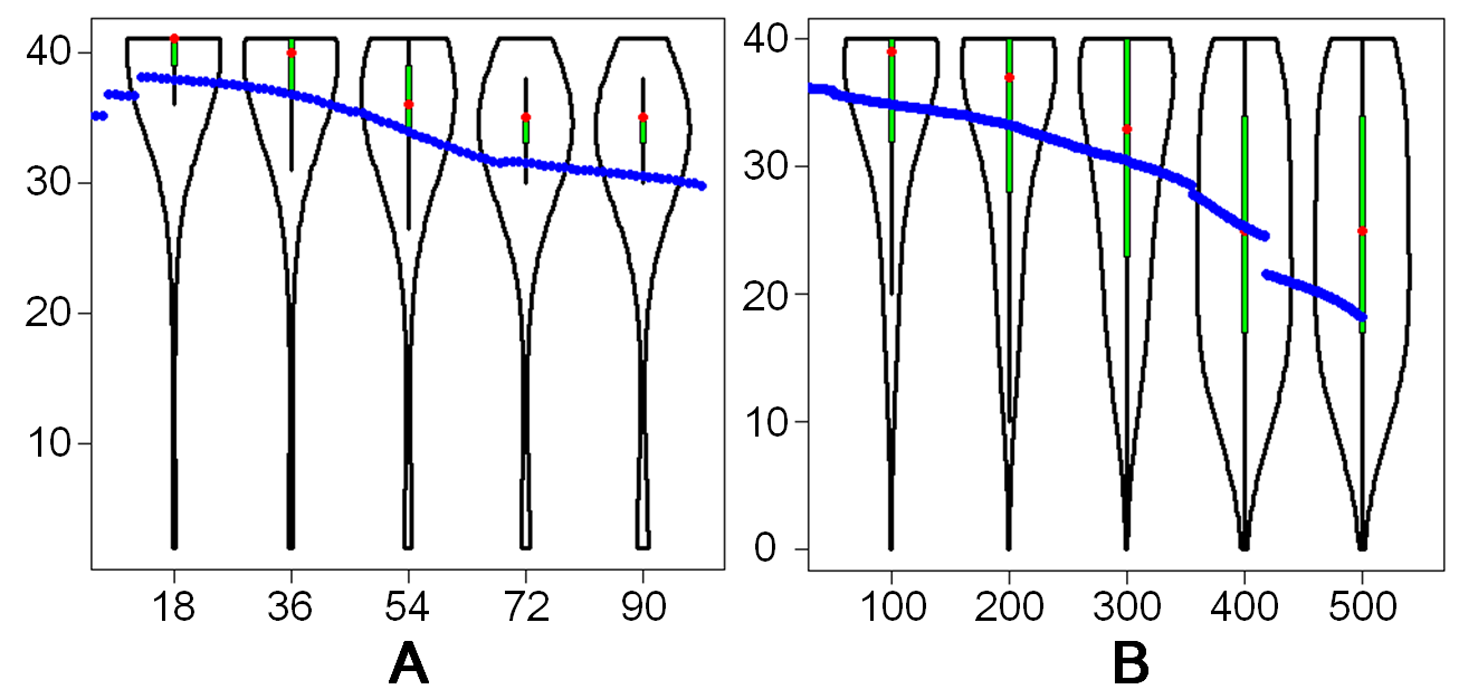

Supplement: Figure S1 — The distributions of quality values at each base plotted by vioplot. (TIF) [file pone.0075448.s001.tif]
