# Supplementary material for: NeSSM: A Next-Generation Sequencing Simulator for Metagenomics
Source: PLoS One. 2013 Oct 4;8(10):e75448. doi: 10.1371/journal.pone.0075448 (PMC3790878; doi:10.1371/journal.pone.0075448)
Supplement: Table S2 — The proportions of substitution errors used in Illumina sequencing simulation. (DOCX) [file pone.0075448.s003.docx]

**Table S2. The proportions of substitution errors used in Illumina sequencing simulation.**

| Substitute  Real | A | T | C | G |
| --- | --- | --- | --- | --- |
| A |  | 0.0389 | 0.11381 | 0.1047 |
| T | 0.03727 |  | 0.10377 | 0.11207 |
| C | 0.11506 | 0.09144 |  | 0.03755 |
| G | 0.09239 | 0.11495 | 0.03809 |  |
