# Supplementary material for: NeSSM: A Next-Generation Sequencing Simulator for Metagenomics
Source: PLoS One. 2013 Oct 4;8(10):e75448. doi: 10.1371/journal.pone.0075448 (PMC3790878; doi:10.1371/journal.pone.0075448)
Supplement: Table S5 — The comparison of the proportions of different kinds of substitutions before and after simulation in Dataset F. The layout of this table is similar to that of Table 3. (DOCX) [file pone.0075448.s006.docx]

**Table S5. The proportions of substitution errors used in Illumina sequencing simulation.**

|  | A | T | C | G |
| --- | --- | --- | --- | --- |
| A | **$\frac{0}{0}$** | **$\frac{0.08497}{0.08311}$** | **$\frac{0.11260}{0.10977}$** | **$\frac{0.09300}{0.09075}$** |
| T | **$\frac{0.08395}{0.08262}$** | **$\frac{0}{0}$** | **$\frac{0.09300}{0.09198}$** | **$\frac{0.11222}{0.11142}$** |
| C | **$\frac{0.07659}{0.07858}$** | **$\frac{0.07427}{0.07667}$** | **$\frac{0}{0}$** | **$\frac{0.05937}{0.06109}$** |
| G | **$\frac{0.07304}{0.07473}$** | **$\frac{0.07809}{0.07932}$** | **$\frac{0.05892}{0.05996}$** | **$\frac{0}{0}$** |
